# Successful treatment of cortical visual impairment in children using anti-amblyopia treatment despite the absence of amblyopia: a case report

**DOI:** 10.1186/s13052-024-01679-w

**Published:** 2024-07-02

**Authors:** Attilio Sica, Paola Michieletto, Stefano Pensiero, Egidio Barbi

**Affiliations:** 1 University of Trieste, Via dei Piccardi, 23, Trieste, 34141 Italy; 2 Institute for Maternal and Child Health - IRCCS “Burlo Garofolo”, Trieste, Italy

**Keywords:** Cortical visual impairment, Anti-amblyopia therapy, Hypoxic-ischemic injury, Multidisciplinary rehabilitation, Developmental approach

## Abstract

**Background:**

Cortical visual impairment (CVI) is a verifiable visual dysfunction that cannot be attributed to disorders of the anterior visual pathways or any potentially co-occurring ocular impairment. Given the limited knowledge on the most effective interventions for visual impairment resulting from CVI, this case report provides valuable insights into an example of successful implementation of anti-amblyopia therapy in a patient with CVI.

**Case presentation:**

This case report presents a 5-year-old girl with CVI secondary to hypoxic-ischemic injury, resulting in visual impairment, dyspraxia, and abnormal visual evoked potential testing. The girl did not suffer from amblyopia, there was no evidence of relevant refractive errors or strabismus, so visual pathway damage was the cause of her visual deficit. Nevertheless, the patient underwent anti-amblyopia therapy and showed significant improvement in visual acuity after 12 months of treatment. The improvement, resulting from visual stimulation, was due to a good functional recovery by a better usage of the damaged visual pathways. The therapy included prescribing corrective glasses and implementing secondary occlusion of the better eye for 4 months, which was protracted for another 4 months, leading to further improvements in visual acuity.

**Conclusions:**

The case report shows that addressing even minor refractive errors and implementing anti-amblyopia therapy can significantly improve vision in children with CVI, even without co-existing amblyopia. It also highlights the importance of early intervention and multidisciplinary rehabilitation in children with CVI, focusing on motor and cognitive skills. Additionally, it emphasizes the need for further research to establish evidence-based practice standards for improving vision in children with CVI.

## Background

Cortical visual impairment (CVI) is a verifiable visual dysfunction that cannot be attributed to disorders of the anterior visual pathways or any potentially co-occurring ocular impairment [1]. To date, no intervention for CVI has been shown to be a high-quality, evidence-based intervention and the approach to CVI treatment does not commonly include anti-amblyopia strategies [2]. Here we present the case of a 5-year-old girl with CVI referred to our ophthalmology clinic for visual impairment who was successfully treated by the implementation of anti-amblyopia therapy, in spite of the absence of amblyopia.

## Case presentation

Our patient was born from a heterozygous twin delivery, with a gestational age of 37 + 3 and a birth weight of 2.400Kg (10th percentile), a length of 46.5 cm (8th percentile), and a head circumference of 32.5 cm (6th percentile). The mother had a history of preeclampsia during pregnancy. The baby had an APGAR score of 5 at one minute of life, which improved to 7 at five minutes and 9 at ten minutes of life. Further evaluation showed no other significant findings, and the baby’s vital signs remained stable until discharge. The patient’s twin sister has no ophthalmological diseases or other health problems.

At her first evaluation at our ophthalmology clinic, the patient, who had just turned 5 years old, had a visual acuity measured to be 2/10 *(20/100)* in the right eye and 5/10 *(20/40)* in the left eye. Refraction was characterized by a moderate bilateral compound hypermetropic astigmatism and there was no evidence of strabismus. Anterior segment and fundus examinations were normal, as well as Optical Coherence Tomography (OCT) of macular and optic disc regions. The OCT of the macula showed no signs of foveal hypoplasia. As concerns the optic disc, the retinal nerve fiber layer (RNFL) was within normal limits for age. Right eye measurements were: superior region 108 µm; inferior region 106 µm; nasal region 70 µm; temporal region 74 µm. Left eye measurements were: superior region 108 µm; inferior region 102 µm; nasal region 67 µm; temporal region 69 µm. These values, along with the normal appearance of the optic disc, confirm the absence of optic nerve hypoplasia or atrophy. Visual evoked potential (VEP) testing revealed increased latency in the right eye compared to the left eye, with an abnormal pattern: the pattern-VEPs (15’) presented a latency of 126 ms and an amplitude of 5 µV in the right eye and a latency of 110 ms and 8 µV in the left eye; this latency difference was present, but to a lesser extent, also in the flash VEPs (120 ms in the right and 116 ms in the left eye). The patient was also noted to have difficulty with gross and fine motor coordination, and her mother described her as “clumsy” compared to her twin sister. A neurological examination, conducted a few days after the initial assessment, revealed dyspraxia with no discrepancies between the right and left sides, accompanied by difficulties in eliciting extrinsic eye movements. However, her language skills, evaluated through the administration of the Test Fono Lessicale (TFL) [3], were well-structured with appropriate content and good communicative intention.

**Fig. 1:**
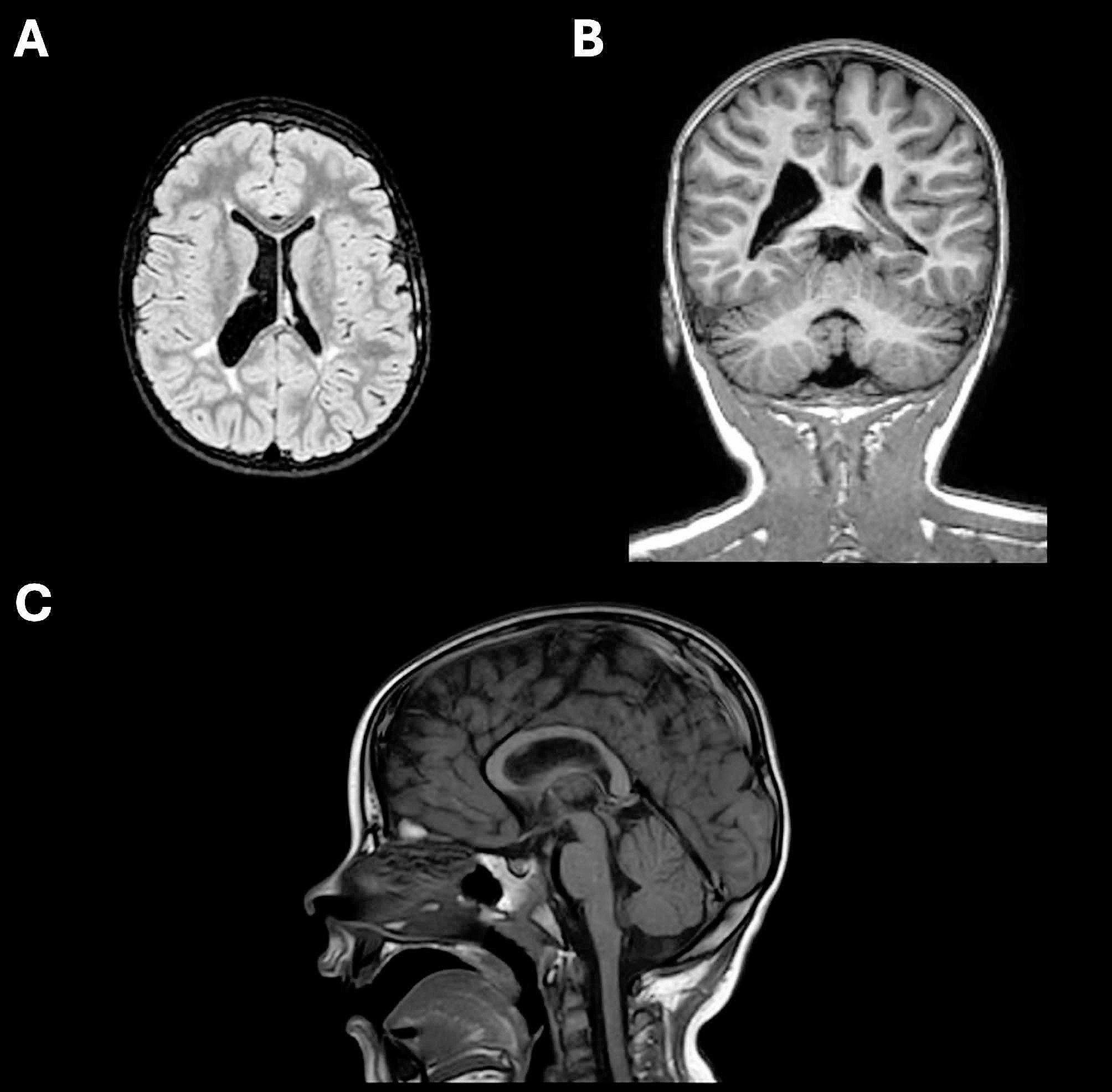
Encephalic MRI scans comprehensive of a T2-FLAIR axial view (A), a T1-weighted coronal view (B), and a T1-weighted sagittal view (C). Neuroimaging reveals asymmetrical enlargement of the right ventricle, slight enlargement of the periencephalic liquor spaces in the left temporal pole, periventricular white matter thinning and gliosis, and corpus callosum abnormalities in posterior brain regions

Three weeks later, a brain MRI was performed, which showed asymmetric enlargement of the lateral ventricles, mild enlargement of the periencephalic liquor spaces in the left temporal pole and thinning and gliotic hyperintensity of the periventricular white matter in the posterior region, mainly on the right side. The biometric values of the corpus callosum (CC) compared with the reference values for 5-year-old girls [4] showed a slight thinning in the posterior region before the splenium. Specifically, the anteroposterior diameter of the CC measured 55.3 mm (< 3rd percentile). Similarly, the thicknesses of the genu and the splenium of the CC were near the 3rd percentile at 6.63 mm and 6.46 mm, respectively. Measurements for the body and isthmus of the CC were 5.10 mm and 4.31 mm, while the fronto-occipital diameter was 155 mm (Fig. 1).

Therefore, visual impairment was caused by cerebral damage, resulting from a mild-grade perinatal hypoxic-ischemic encephalopathy (Stage 1 of Sarnat & Sarnat, 1976) [5], involving the brain’s visual regions and optic radiations. It was not caused by asymmetric bilateral amblyopia, which can be due to strabismus or bilateral refractive errors.

At the following evaluation, performed one week later, glasses were prescribed with a non-cycloplegic correction: +0.50 sph + 0.50 cyl80 in the right and + 0.50 sph + 075 cyl 100(TABO) in the left eye. This was done to improve visual acuity in the hypothesis of a coexisting refractive amblyopia due to mild astigmatism alongside neurological damage. After 4 months, visual acuity improved only in the left eye which reached 7/10 *(20/28)*. Following the rehabilitation protocol for bilateral refractive amblyopia, occlusive treatment was initiated through secondary occlusion [6]: 3 h of patching of the left eye daily for 4 months was prescribed. Occlusion was combined with visual stimulation using a digital tablet, either through watching animated cartoons or by engaging in drawing and coloring activities. For the labile eye-hand coordination and a tendency to get easily distracted a rehabilitation program to improve praxis was associated.

After another 4 months, the patient’s visual acuity improved to 4/10 *(20/50)* in the right eye while it was stable in the left eye. Thus, occlusion therapy was protracted for another 4 months, leading to further improvements in visual acuity, which reached 6/10 *(20/32)* in the right eye and 8/10 *(20/25)* in the left eye. There was no further improvement in the following 4 months.

In summary, although the patient’s visual impairment was caused by CVI, a significant improvement was achieved after 12 months of therapy. A maintenance therapy, involving 1 h of occlusion per day, was carried out over the following 6 months, with the final visual acuity remaining unchanged.

The Developmental Test of Visual Perception 2nd Edition (DTVP-2) was used to assess the impact of improved visual acuity and motor rehabilitation on eye-hand coordination. Two subtests of the DTVP-2 were administered to the child twice, at 5 and 6 years: the eye-hand coordination subtest and the copying subtest. At the first examination the girl achieved a score of 48 (5th percentile) in the first subtest and a score of 2 (9th percentile) in the second one. One year later, her scores improved significantly to 140 (37th percentile) and 13 (25th percentile) respectively in the same subtests. This marked improvement indicates a substantial enhancement in the child’s visuomotor abilities, exceeding typical developmental progress associated with age.

## Discussion and conclusions

In this case report, the patient presented with visual impairment and dyspraxia, but did not meet the criteria for asymmetric refractive bilateral amblyopia. This condition is typically defined as best-corrected visual acuity of 20/40 or worse in both eyes with an interocular difference of two lines or more, in the presence of 4.00 diopters (D) or more of hypermetropia by spherical equivalent, 2.00 D or more of astigmatism, or both in each eye [7]. Additionally, the patient displayed abnormal results on VEP testing. This prompted further evaluation with a brain MRI, which revealed structural abnormalities in the brain consistent with CVI secondary to hypoxic-ischemic damage.

Many works show how MRI-detectable changes can provide valuable insights into clinical outcomes, reveals significant brain abnormalities that correlate with specific neurological manifestations [8]. In prematurely born infants, MRI-detected alterations in the corpus callosum, cingulum, anterior commissure, fornix, and right uncinate fasciculus have been reported to correlate with eye-hand coordination but not linguistic performance [9]. Conversely, impaired linguistic performance has been associated with disruptions in the frontal region, particularly the ventral precentral gyrus and the arcuate fasciculus [10]. In our case report, the impairment in eye-hand coordination can be ascribed to the alterations in the corpus callosum, and possibly in the right uncinate fasciculus (given the extent of the anterior dilation of the right lateral ventricle) detected on MRI.

CVI is the leading cause of visual impairment in children in the USA [11] and in Europe [12, 13]. Although current practice standards recommend early intervention [2] on motor and cognitive skills, little is known about which interventions are best suited to vision and there is currently no standardized treatment for CVI. The only randomized trial was conducted in 1991 and indicates that a developmental approach to early vision promotion leads to faster improvements in all vision functions [14]. Several confounding variables, such as the coexistence of other neurological and ophthalmological anomalies and the inherent processes of children’s physiological development, make it challenging to ascertain accurate correlations between interventions and outcomes [2]. However, timely interventions, including modifications to the visual environment, comprehensive multidisciplinary care and surgical procedures, have been shown to be beneficial in treating ocular and systemic comorbidities in affected children, supporting an effective rehabilitative process [15, 16].

Typical interventions in patients with CVI encompass visual stimulation techniques for both eyes, such as exposure to light, patterns, or slides [17], and strategic environmental modifications, such as simplifying the visual environment, minimizing crowding, and using objects with distinct colors, high contrast, and motion to facilitate visual recognition. Infants and toddlers under the age of two are generally the only ones targeted by these treatments. However, empirical outcomes have been variable, indicating that children diagnosed with CVI may achieve improvements with or without the application of visual stimulation strategies.

In the case study presented, the intervention was implemented at a later stage—at five years of age—yet still within the critical period of neural plasticity. The efficacy of the anti-amblyopic approach employed demonstrated the nervous system’s substantial capacity for adaptation and recovery, a phenomenon that underscores the broader principle of brain plasticity, where structural changes in brain circuitry or modifications of the synaptic connections can happen in response to variations of environmental stimuli [18]. Such plasticity, which diminishes with age, becoming mild after 8–10 years, has also been demonstrated in cases of childhood brain injuries, including cases of hypoxic ischemic encephalopathy [19].

In our case report, concurrent multidisciplinary rehabilitation has allowed for a significant improvement in motor skills as well. In fact, even a small correction of astigmatism has led to an improvement in the visual acuity of the better eye in just 4 months, while occlusion allowed a significant improvement in the worse eye within 8 months, periods too short to justify the improvement as a result of a developmental process in a child already 5 years old. Furthermore, given the absence of strabismus or significant refractive defects, these improvements cannot be due to amblyopia recovery but to a better use of the visual pathway, even if damaged.

In conclusion, this case report illustrates the successful implementation of anti-amblyopia therapy in a child with CVI (Table 1) and allows us to affirm that it is necessary to correct all refractive defects, even if of little amount, and to implement all possible therapies (occlusion, macular stimulations, and others) to improve the vision of these children, in the context of multidisciplinary rehabilitation. Further studies will be needed to understand the potential for broader application of anti-amblyopia therapy in similar cases, which could be crucial in defining treatment protocols and ultimately enhancing the quality of life for children affected by CVI.

**Table 1:** Case report timeline

Dates	Relevant past medical history and interventions		
Birth	Mother had preeclampsia during pregnancy Gestational age: 37 + 3 weeks. Birth weight: 2.400 kg (10th percentile) APGAR scores: 5 at one minute, 7 at five minutes, 9 at ten minutes Vital signs stable, no significant health findings, and discharge from hospital		
**Dates**	**Summaries from Initial and Follow-up Visits**	**Diagnostic Testing**	**Interventions**
Time 0 (Age 5)	Visual acuity measured: 2/10 in the right eye and 5/10 in the left eye Moderate bilateral compound hypermetropic astigmatism Normal OCT VEP: Increased latency in right eye (126 ms) compared to left eye (110 ms)	Initial evaluation OCT VEP testing	
1 week	Diagnosis of dyspraxia with normal language skills Visual & Motor Assessment shows low percentiles in visuomotor abilities	Neurological examination DTVP-2	
1 month	MRI reveals asymmetrical enlargement of the right ventricle, enlargement of the periencephalic liquor spaces, periventricular white matter thinning and gliosis, and corpus callosum abnormalities	Brain MRI	Prescribed glasses: - right eye: +0.50 sph + 0.50 cyl80 - left eye: +0.50 sph + 075 cyl 100(TABO)
5 months	Visual acuity improved in left eye (7/10)		Started occlusive treatment for left eye
9 months	Visual acuity improved in right eye (4/10); stable in left eye		Continuation of occlusive therapy
13 months	Further improvement in visual acuity of right (6/10) and left (8/10) eyes Visual & Motor Assessment shows significant improvement in percentiles	DTVP-2	Maintenance occlusive therapy
19 months	Final visual acuity remained unchanged		

*Chronological overview of the medical visits, diagnostic evaluations, and therapeutic interventions performed*

## Data Availability

Not applicable.

## Abbreviations

CC: Corpus Callosum
CVI: Cortical Visual Impairment
cyl: Cylinder
D: Diopters
MRI: Magnetic Resonance Imaging
OCT: Optical Coherence Tomography
VEP: Visual Evoked Potential
sph: Spherical Equivalent
TABO: Ophthalmological Abbreviations Table (Tabelle di Abbreviazioni Oftalmologiche)
